# Pain perception in autism. A study of sensory reactivity in children and adolescents with autism using quantitative sensory testing and psychophysiological correlates

**DOI:** 10.3389/fnins.2025.1543538

**Published:** 2025-05-01

**Authors:** Valentina Nicolardi, Isabella Fanizza, Giuseppe Accogli, Sara Scoditti, Antonio Trabacca

**Affiliations:** ^1^Scientific Institute IRCCS “E. Medea”, Bosisio Parini, LC, Italy; ^2^Associazione “La Nostra Famiglia” – IRCCS “E. Medea” - Scientific Hospital for Neurorehabilitation - Unit for Severe Disabilities in Developmental Age and Young Adults (Developmental Neurology and Neurorehabilitation), Brindisi, Italy

**Keywords:** pain, QST, EEG, autism, ERPs, neurodevelopment, perception

## Abstract

**Background:**

Hyper- or hypo-reactivity to sensory input is a diagnostic criterion for autism spectrum disorder; however, it is still not fully characterized, despite its relevance to patients' quality of life. When considering neurodevelopment, sensory reactivity in autism is often assessed through parental reports, with only a few pieces of evidence acquired using standardized protocols. Quantitative sensory testing (QST) is a standardized protocol used to quantify sensory function by assessing perceptive and pain thresholds with calibrated sensory stimuli. To date, only a few studies have used QST to investigate sensory reactivity in autism, with only one taking into account adolescents and none including children in the sample.

**Methods:**

We aimed to study pain perception and in children diagnosed with autism using the QST protocol. Moreover, we sought to measure central reactivity to painful stimuli by recording electroencephalographic (EEG) responses to painful thermal stimuli to explore the relationship between subjective reactivity (i.e., reactions to sensory stimuli) and central processing of sensory stimuli (i.e., EEG responses). Finally, we aimed to explore the relationship between parents' reports, subjective reports, and EEG responses.

**Discussion:**

This study will help to expand our previous knowledge concerning the sensory profile of children and adolescents with autism. Deepening our understanding of the relationship between perceptive thresholds in children with autism and the reactivity of the central nervous system, could help us understand the causal mechanism of the perceptual differences observed in autism.

**Study protocol identifier:**

NCT06659731

## 1 Introduction

Since 2013, the updated diagnostic criteria for autism, which include “hyper- or hypo-reactivity to sensory input or unusual interests in sensory aspects of the environment,” have been in place (American Psychiatric Association, 2022). In children, these two sensory profiles have been mostly studied through parental self-reports, such as the Short Sensory Profile (McIntosh et al., 1999) and the Short Sensory Profile II (Dunn, 2014). Recent studies based on these measures have reported different clusters of results, dividing children into two (Simpson et al., 2019) or five phenotypes (Scheerer et al., 2021). Simpson et al. (2019) found two clusters using the SSP-2: one cluster named *Uniformly elevated*, which reported high scores across all quadrants, and another cluster named *Raised avoiding and sensitivity*, which showed higher scores in the avoiding and sensitivity quadrants. More recently, Scheerer et al. (2021), using the SSP, identified five distinguished sensory profiles that may account for some of the behavioral heterogeneity in people with autism. Such heterogeneity was also highlighted by Proff et al. (2022) in a recent review focused on sensory processing in autism, particularly in the exteroceptive and interoceptive domains. This finding highlights that accounting for the heterogeneity of sensory processing is crucial for understanding and diagnosing autism, as well as for treatment and fostering inclusivity in our society. Pain perception is another domain of perception affected by autism. Patients may be at higher risk of self-injurious behaviors (Rattaz et al., 2013) or may have issues communicating the pain they experience (Fitzpatrick et al., 2022). Moreover, pain and its chronicity represent actual public health issues (Blyth, 2008; Cohen et al., 2021), especially for clinical populations, such as people with autism. Recently, Moore (2015) highlighted the need for a systematic investigation of pain in autism through standardized and more reproducible methodologies. Previously, it has been reported that different studies have considered various outcome measures, such as parent reports, self-reports, sensory thresholds, and objective measures. This variability has reduced consistency across different studies and results. Moreover, the absence of control groups and the use of non-standard pain measures have contributed to conflicting results, according to Moore (2015). Recently, we reviewed all the studies published after Moore (2015) review that used quantitative sensory testing (QST) to study pain in autism, and we found that only five studies investigated pain using the QST methodology. Of these few studies, only one considered adolescents, and none considered children (Nicolardi et al., 2023). This literature gap could reasonably be due to the amount of discomfort possibly associated with the administration of sensory stimuli to children and adolescents with autism, which could lead to a high number of dropouts within the sample. With a standardized protocol, such as QST, the challenge of reproducing the methodology is further increased by the necessity to test both the left and right sides and by the overall protocol duration. Indeed, the few studies that have approached this topic using QST have implemented a modified version suitable for patients' difficulties (Symons et al., 2022; Barney et al., 2020). Even when the same methodology was used (QST), different hypotheses, outcomes, and samples were considered. Two studies (Fründt et al., 2017; Vaughan et al., 2020) reported differences in the perception of mechanical stimuli in autism. However, other studies found lower sensitivity to thermal stimuli in people with autism (Duerden et al., 2015; Chien et al., 2020). Chien et al. (2020) also collected other measures alongside QST, reporting a decrease in sensory fiber density in a sub-sample, which could explain the sensory features in this specific sample. The most recent study concerning QST to assess sensory perception in autism involved 52 adults diagnosed with autism (Hoffman et al., 2023). The authors concluded that the clinical sample reported hypersensitivity to daily stimuli and experimental pain, with less inhibition of tonic painful stimuli.

Considering the methodological limitations highlighted by previous literature and the need for a standardized and more reproducible methodology, we decided to use the QST protocol to investigate pain perception in children and adolescents with autism. We integrated this measure with an assessment of the central processing of painful stimuli, collected through electroencephalographic (EEG) measures. Finally, we compared the data from this sample with data from an age-matched control sample. Through this combination of QST measures and EEG measures, we aimed to deepen the characterization of the sensory profile and pain perception in autism, expanding the general understanding of the peripheral and central contributions of sensory hyper- or hypo-reactivity in autism across the neurodevelopmental stages.

## 2 Methods and analysis

**Figure 1:**
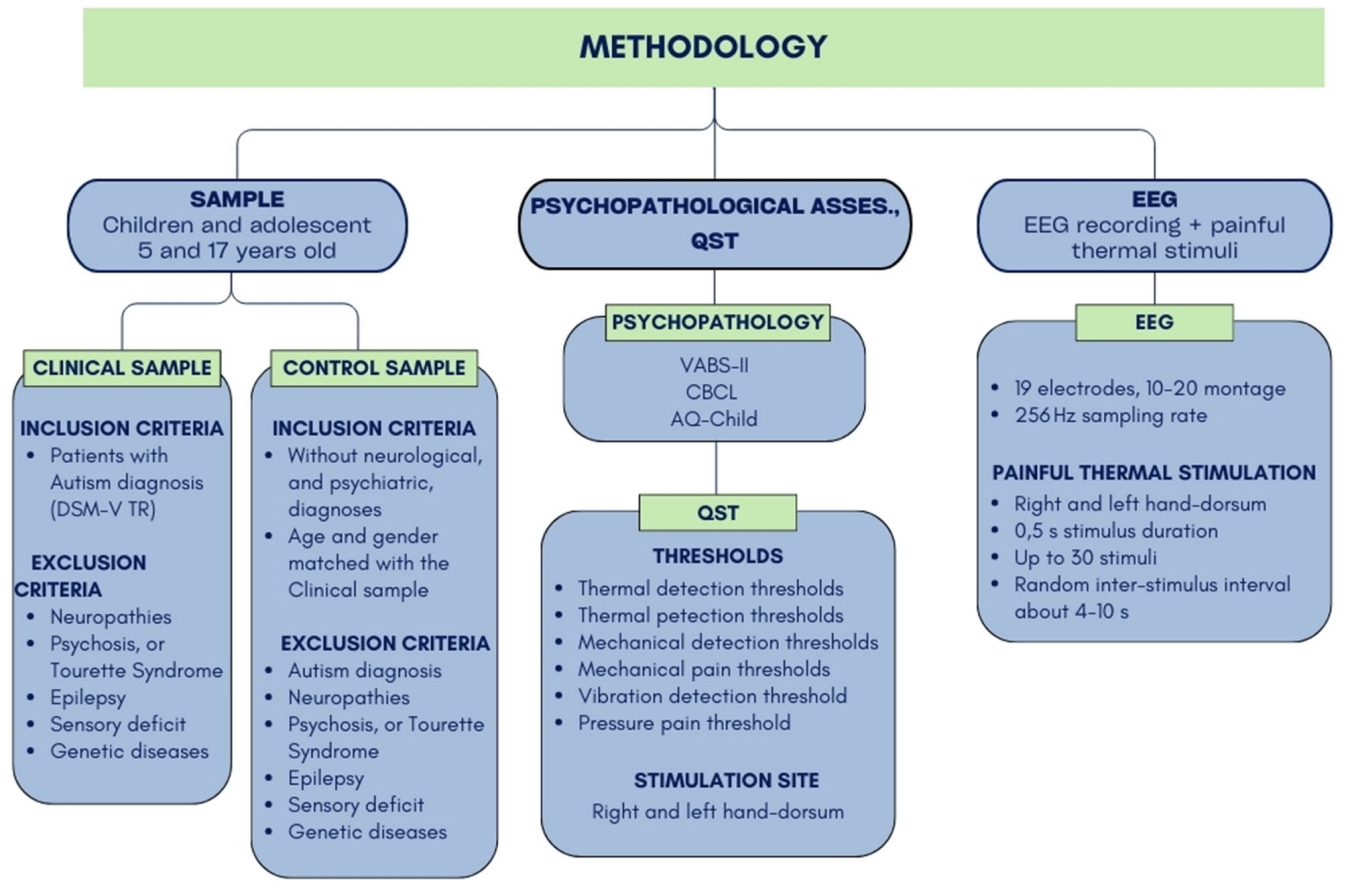
Left boxes (SAMPLE): the inclusion and exclusion criteria for the clinical and control sample. Central boxes (PSYCHOPATHOLOGICAL ASSESS., QST): the list of psychopathological assess scales and the thresholds measured through the QST procedure. Right boxes (EEG): EEG recording and thermal stimulation features.

This was an observational, case–control clinical study. The study procedure involved the following steps (Steps 2 to 4 shown in Figure 1): (1) Eligibility assessment according to the inclusion and exclusion criteria, (2) Administration of questionnaires and scales for the characterization of clinical aspects of autism: the Short Sensory Profile (Tomchek and Dunn, 2007), the Autism Spectrum Quotient—Children's Version (AQ-Child. Auyeung et al., 2008), the Vineland Adaptive Behavior Scales—Second Edition (VABS-II; Sparrow et al., 2005), and the Child Behavior Checklist (CBCL) (Achenbach and Rescorla, 2001), (3) Administration of modified QST to study sensory thresholds and perception, and (4) EEG recording at rest and while receiving heat stimuli to study cortical processing of salient thermal stimuli.

### 2.1 Sample

The sample included children and adolescents between the ages of 5 and 17 years selected according to the following inclusion/exclusion criteria. According to the calculation performed with the G^*^Power software, the sample size should be approximately 32 participants to provide a statistical power of 0.9. The sample size calculation was performed based on the effect-size parameters by Vaughan et al. (2020).

*Inclusion criteria for the clinical sample*

Children and adolescents between the ages of 5 and 17 years and patients with autism diagnosis according to the DSM-V TR criteria were included in the clinical sample.

*Exclusion criteria for the clinical sample*

Patients with the following comorbidities were excluded from the clinical sample: peripheral neuropathies, to avoid confounding factors with respect to the QST measures; psychiatric diagnoses, such as psychosis or Tourette syndrome, to prevent interference with EEG signals; neurological diagnoses such as epilepsy, sensory deficits, or sensory loss; and genetic diseases.

*Inclusion criteria for the control sample*

The inclusion criteria include the following: Healthy children and adolescents between the ages of 5 and 17 years, without neurological or psychiatric diagnoses and age- and gender-matched participants within the clinical sample.

*Exclusion criteria for the control sample*

Children and adolescents with an autism diagnosis and those with peripheral neuropathies; psychiatric diagnoses such as psychosis or Tourette syndrome, to prevent interference with EEG signals; neurological diagnoses such as epilepsy, sensory deficits, or sensory loss; and genetic diseases were excluded.

### 2.2 Psychopathological assessment and dispositional measures

The psychopathological assessment (Figure 1) allowed for the characterization of clinical aspects of autism through the following scales: the Vineland Adaptive Behavior Scales—Second Edition (VABS-II; Sparrow et al., 2005); the Child Behavior Checklist (CBCL; Achenbach and Rescorla, 2001); and the Autism Spectrum Quotient—Children's Version (AQ-Child; Auyeung et al., 2008). Moreover, a caregiver report questionnaire was included to assess sensory processing abnormalities in children with autism and neurotypical children: the Short Sensory Profile (Tomchek and Dunn, 2007).

### 2.3 QST

The QST protocol (Rolke et al., 2006; Blankenburg et al., 2010) involves the administration of seven short sensory tests to measure up to 13 parameters. The overall parameters can be grouped as follows (Figure 1): thermal detection thresholds for the perception of cold, warm, and paradoxical heat sensations; thermal pain thresholds for cold and hot stimuli; mechanical detection threshold (MDT) for touch and vibration; mechanical pain threshold (MPT) and sensitivity for pinprick and blunt pressure; and vibration detection and pressure pain thresholds. Previous research using QST in neurodevelopmental disorders used a modified version of the whole protocol to make it suitable and tolerable for different clinical conditions, such as developmental delay, including motor, communicative, and cognitive impairments (Barney et al., 2020; Symons et al., 2022). Thus, we administered the most suitable and tolerable version of the QST based on each patient's condition and needs. We selected the hand dorsum as the only stimulation site and reduced the number of stimuli for each stimulation to shorten the stimulus trial duration and enhance the tolerability of the complete procedure. For each intensity value, three stimuli were delivered on the hand dorsum for mechanical stimulation. We also divided the QST procedure into two sessions, conducted at the same time of day for each patient (i.e., both sessions in the morning or both in the afternoon). The entire procedure was conducted in a child-friendly environment—colorful and comfortable, with sensory stimuli adapted to avoid fearfulness of the stimuli. Thus, each stimulation was presented as a game to be played with a specific animal, and each device and instrument was covered with pictures of animals according to the game it represented. Overall, QST lasted ~1 h 30 min. When needed, it was divided into two sessions, each lasting ~45 min, according to the patients' needs. Our modified version of QST only included detection and pain thresholds for thermal and mechanical (pinprick, vibratory, and pressure) stimuli. We excluded the following from the original procedure: paradoxical heat sensations, the difference limen threshold for alternating cold and warm stimuli, and the number of paradoxical heat sensations, as these stimulations involved many repeated stimuli, greater unpleasantness, and less tolerability for patients. Thermal stimulation included cold and warm detection thresholds (CDT and WDT, respectively) and cold pain and heat pain thresholds (CPT and HPT, respectively). These parameters were estimated using the method of levels, implemented with a contact thermal cutaneous stimulator (TCS model II.1, QST.Lab; Strasbourg, France) to avoid variations in response times related to the method of limits (Duerden et al., 2015; Chien et al., 2020). We chose the thermode model T08, which consists of 15 Peltier elements, each with a 9 mm^2^ surface, resulting in a total stimulation surface of ~4.5 cm^2^. This model was selected as it is the most suitable probe for implementing the QST protocol and contact heat-evoked potentials. The mechanical detection threshold (MDT) was determined using 12 modified von Frey hairs (Optihair2-Set, Marstock Nervtest, Germany), with stimulus intensities of 0.25, 0.5, 1.0, 2.0, 4.0, 8.0, 16.0, 32, 64, 128, 256, and 512 mN. The mechanical pain threshold (MPT) was measured using weighted pinprick mechanical stimulators, with intensity forces of 8, 16, 32, 64, 128, 256, and 512 mN and a contact area of 0.2 mm in diameter. The vibration detection threshold (VDT) was measured using a Rydel-Seiffer graded tuning fork (64 H/.8/8 scale) placed on the styloid process of the ulna. The tuning fork was placed on the target bone and kept in place until the patient could no longer feel the vibration. The pressure pain threshold (PPT) was measured using a calibrated digital force gauge device (SAUTER FC) with a maximum capacity of up to 50 N and a probe area of 1 cm^2^. Concerning possible communicative impairments, we planned to rely on parents' help in understanding the specific communication modalities of each patient, as well as to introduce the use of non-verbal pain scales, such as the Pain and Discomfort Scale (PADS; Shinde et al., 2014), when needed.

### 2.4 EEG recording with thermal stimulation

The EEG signal was acquired using the BRAIN QUICK EEG system (MicroMed, Treviso, Italy) with a 256 Hz sampling rate from 19 electrodes disposed according to the international 10–20 system. The reference electrode was placed on the right earlobe. The EEG recording session began with the cap montage, followed by a short accommodation with the sensory stimuli to determine an intensity value for a moderately painful stimulus to be used for the stimulation. Moderately painful stimulus refers to one that the patient perceives as painful but still tolerable, with low intensity and minimal unpleasantness. This finding ensures the procedure's tolerability and minimizes patient discomfort. After the preparation/accommodation, the EEG recording was performed with the eyes open, starting with a series of up to 30 thermal stimuli (according to the patients' tolerability) delivered to the patients' right-hand dorsum with a random inter-stimulus interval between 4 s and 10 s in the step of 1 s. Stimulus duration was 0.5 s. The entire EEG recording, lasting ~30 min, included an EEG cap montage, instructions, and stimulation, with breaks provided if needed by the patients. We did not include systematic time breaks by default, as the painful stimulation procedure lasted < 10 min. However, given the age of the patients, we accounted for the possibility of breaks and included them in the 30 min time estimation.

### 2.5 Analysis

The primary outcome measures of this study were sensory thresholds: CDT and WDT, CPT and HPT, MDT and MPT, VDT and PPT. Another primary outcome was EEG responses, analyzed in the time domain (ERPs related to the thermal stimulus) and the time-frequency domain (power spectrum, both phase-locked and non-phase-locked with the thermal stimulus). The QST threshold data were processed according to Rolke et al. (2006), while normative values from Blankenburg et al. (2010) were used for z-transformation. Statistical analysis of the primary outcomes involved a one-way ANCOVA, with sex as a covariate, to compare the effect of the group variable (autism/neurotypical controls) on the dependent variables.

The secondary outcome measures of this study were the scores on dispositional and psychopathological measures, which were used for exploratory analysis in relation to the primary outcomes. For the explorative analysis, mixed-effects models were implemented, accounting for inter-individual variability (random intercept), and when possible (according to model convergence), for dispositional and psychopathological measures as grouping factors (random slopes).

The EEG data were processed and extracted using the EEGLAB (Delorme and Makeig, 2004) and Letswave software (www.letswave.org). Signal processing involved artifact removal through a band-pass filter. Ocular artifacts were identified and removed using independent component analysis (ICA) decomposition, implemented with the *runica* algorithm in EEGLAB (Delorme and Makeig, 2004), and re-referenced to the average reference. Further processing included signal averaging for the extraction of event-related potentials (ERPs) in time-domain. According to previous literature and the methodology used, contact-heat evoked potentials (Chien et al., 2020; Mulders et al., 2020) were analyzed and compared between the two groups. Nonetheless, further exploratory analysis was conducted on vertex components typically related to sensory processing (Baumgartner et al., 1993; Mouraux and Plaghki, 2007). Computations, such as a fast Fourier transform or wavelet transform, were implemented to extract time-frequency domain information. Finally, explorative analyses were conducted to clarify if the psychopathological or dispositional measures contributed to group differences in sensory perception or EEG responses. In addition, we explored the relationship between subjective differences and cortical reactivity measured through EEG.

## 3 Discussion

To date, this is the first investigation of the sensory profile of children and adolescents with autism using the QST method. In line with previous results (Duerden et al., 2015; Fründt et al., 2017; Vaughan et al., 2020; Chien et al., 2020), we hypothesized group differences at the perceptive level. We expected to find higher variability in the clinical sample due to the presence of hyper- and hypo-sensitivity within the same sample. Moreover, we also expected to find differences in the EEG measures, indicating different cortical reactivity between the two groups. As for the perceptive profiles and EEG measures, we expected higher variability within the clinical sample. This variability could allow us to further divide this sample based on hyper- or hypo-sensitivity profiles and them as a grouping factor and choose the statistical comparison according to their presence. Moreover, with this protocol, we aimed to overcome a methodological challenge by implementing a standardized clinical protocol with children with autism, a protocol typically performed and validated on neurotypicals. The challenge concerns not only the methodology *per se* but also the topics of inclusivity and neurodiversity in research. Indeed, we adapted the protocol according to the differences and needs of the individuals with autism, increasing the protocol's tolerability for the patients. We followed the guidelines from previous studies on QST in children with intellectual and developmental disabilities (Blankenburg et al., 2018; Symons et al., 2022; Barney et al., 2020). Previous studies modified the original QST protocol to make it more tolerable in terms of the number of stimuli, time duration, and communication (Symons et al., 2022; Barney et al., 2020). Some parts of the protocol could be uncomfortable and possibly overwhelming for patients, such as the difference limen for cold and warm stimuli, paradoxical heat sensation, and dynamic mechanic allodynia, which may be due to the alternation of different stimuli in a very short time, as well as the intense, repetitive, and fast stimulation that characterizes these parts of the QST protocol. Thus, in line with the methodological aims, we decided not to administer these sessions and to collect only perceptive and pain thresholds. Moreover, we decided to stimulate both sides of the body with only three consecutive stimuli for each intensity value, except for the thermal stimuli, where the number of stimuli was determined by the threshold algorithm of the thermal stimulator. Recent findings have shown between-group differences for mechanical thresholds (Fründt et al., 2017; Vaughan et al., 2020) and thermal thresholds (Duerden et al., 2015; Chien et al., 2020), reporting lower sensitivity for people with autism. Moreover, Chien et al. (2020) found that a subsample exhibited lower skin fiber density and higher amplitude for evoked potentials related to thermal stimuli. The authors suggested that peripheral fiber density could partially explain these results; however, only Chien included this measure in their methods. Finally, the study by Duerden et al. (2015) found lower sensitivity to thermal stimuli in people with autism. The authors used the method of limits to measure detection thresholds, which relies on participants' reaction times. Indeed, the authors suggested that their results could reflect slower response times in the population with autism. On the other hand, Cascio et al. (2008) summarized findings on tactile thresholds in adults with autism, reporting hypersensitivity compared to neurotypical controls. The methodological differences could account for conflicting results; however, it is also possible that these results reflect the higher variability within the clinical sample, where some patients show hyper-sensitivity and others show hypo-sensitivity to sensory stimuli. We claim that the stimulation protocol, the psychophysical approach, and the statistics implemented should be considered to improve the methodological approach concerning perception in autism. In line with this finding, we believe that our study could provide a robust and reproducible methodology and positively contribute to the current literature. The use of QST as the stimulation protocol, a psychophysical approach accounting for differences in reaction times, and a statistical approach that also explores inter-individual differences and grouping factors can overcome previous methodological issues. Increasing our understanding of differences in sensory reactivity and perception by considering subjective, central, and peripheral measures is crucial for planning adequate and personalized clinical intervention (Smith, 2019). Moreover, this methodological approach could contribute to future hypotheses on the causal mechanisms of observed differences. Nonetheless, the use of a non-validated modified version of QST and the sample size could be considered methodological limitations. However, the modifications we applied to the protocol align with previous research on neurodevelopmental disorders, where differences in sensory reactivity were observed when compared to typically developing children (Symons et al., 2010, 2022). If confirmed, our results could align with findings from other neurodevelopmental disorders. Within the framework of inclusivity toward neurodiversity, it is essential to adapt research methodologies that are often applied for neurotypicals. For this reason, we collaborated with patient and family organizations, discussing our methodology with them in line with the participatory research framework.

## Data Availability

The collected data will be made available by the authors, without undue reservation. Further inquiries can be directed to the corresponding author.
